# Region specific microstructural complexity of the ovine meniscus root provides an organizational basis for injury susceptibility

**DOI:** 10.1038/s41598-025-20667-6

**Published:** 2025-09-24

**Authors:** Marta Carretero-Hernández, Yin Zhang, Wei Liu, Magali Cucchiarini, Henning Madry

**Affiliations:** Center of Experimental Orthopaedics, Saarland University, 66421 Homburg, Germany

**Keywords:** Medial meniscus anterior root, Enthesis, Sheep, Type-I collagen, Type-II collagen, Type-X collagen, Bone, Cartilage, Ligaments

## Abstract

Comprehensive information on the meniscus root microstructure is essential to exactly understand its physiological role and susceptibility to injury. We selected the ovine medial meniscus anterior root (MAR) as model to elucidate the intricate spatial arrangement of its enthesis, root ligament and transition into the medial meniscus anterior horn (MMAH), hypothesizing that its microstructure is comparable to humans. We applied different histological, type-I, -II, and -X collagen immunohistochemical, polarization and confocal analyses to investigate its structural complexity. The results reveal unique region-specific patterns. Cell morphology, proteoglycan, and type-II collagen contents differ between regions. The enthesis is avascular while the MAR ligament and red-red zone of the MMAH are well vascularized. The ovine MAR attachment constitutes an enthesis organ together with a bare area below the root ligament covered by adipose tissue. The MAR ligament comprises large longitudinal fascicles that unweave into a complex network when entering the MMAH, changing their orientation towards its white-white zone. The blood vessels that vascularize the MAR ligament enter at its peripheral-femoral side. Only axial MMAH fibers are immunopositive for type-X collagen. This region-specific microstructural complexity of the ovine MAR is largely similar to published findings in humans, providing an organizational basis for injury susceptibility. Thus, the ovine MAR may serve to study the physiopathology of and therapeutic approaches to human root tears.

## Introduction

The meniscus roots are important ligament-like structures in the knee that anchor the menisci to the tibial plateau^1^. It consists of a ligamentous part (root ligament), its fibrocartilaginous insertion into the tibial plateau, and a transition line when entering the meniscus horns. The root enables the meniscus to transfer the load from the femoral condyle to the tibia, to convert compressive load into hoop strain, and to stabilize the knee^2^. Meniscus root tears are defined as soft tissue or bony avulsion injuries or radial tears of the meniscus horn within 10 mm of the root attachment^3^. Root tears severely alter the knee biomechanics, leading to meniscus extrusion^4,5^, articular cartilage degeneration and osteoarthritis (OA)^6,7^.

Meniscus root repairs are increasingly being performed to delay the onset of OA^8^. However, the regeneration of the root anatomy presents challenges, affecting the durability of root repair and ultimately knee function over time^9–13^. Rabbit^14–16^, goat^17^ and sheep^18^ models have been employed to study meniscus root repairs, indicating incomplete microstructural healing^14–17^. Meniscus root anatomic characteristics affect the development of root tears and their regenerative potential for healing or repair. Information about meniscus root microstructure is therefore useful to elucidate the morphological basis and the pathogenesis of root tears in humans^19^ and animal models^17,18,20–22^. Also, analyzing the microstructural anatomy of the meniscus root offers valuable insights that may be also of help to improve the basic science of meniscus root repairs. One of our aims is to develop a preclinical sheep model of medial meniscus anterior root (MAR) repair for translational meniscus^18,22^ and OA research^23,24^. However, no sufficiently precise knowledge exists of the zone-dependent microstructural anatomy of the sheep MAR. Such information is of paramount importance for a correct understanding of its physiological role in the knee joint and the mechanisms of meniscus root repair.

The objective of the present study was to comprehensively analyze the region-specific microstructural complexity of the entire ovine MAR as a potential translational model to address the fundamental question whether it structurally reflects the situation in humans. We hypothesized that the anatomical zonal heterogeneity of the ovine MAR is comparable to that of a human MAR. To verify this, our first goal was to provide an accurate investigation of the regional characteristics of this intricate structure. We defined the distribution of type-I, -II, and -X collagen, proteoglycans, networks of tensile and elastic fibers, vascularization, densities and shapes of cells applying immunostaining, safranin-O/fast green (safranin O), hematoxylin and eosin (HE), picrosirius red, Masson-Goldner trichrome (Masson-Goldner) staining, polarization and confocal microscopic immunofluorescent analysis and compared these findings with published data on the human MAR. Our second goal was to associate the structural findings with the clinical situation, placing an emphasis on the different root injury types.

## Results

### Microstructural analysis of the bare area below the MAR

**Fig. 1:**
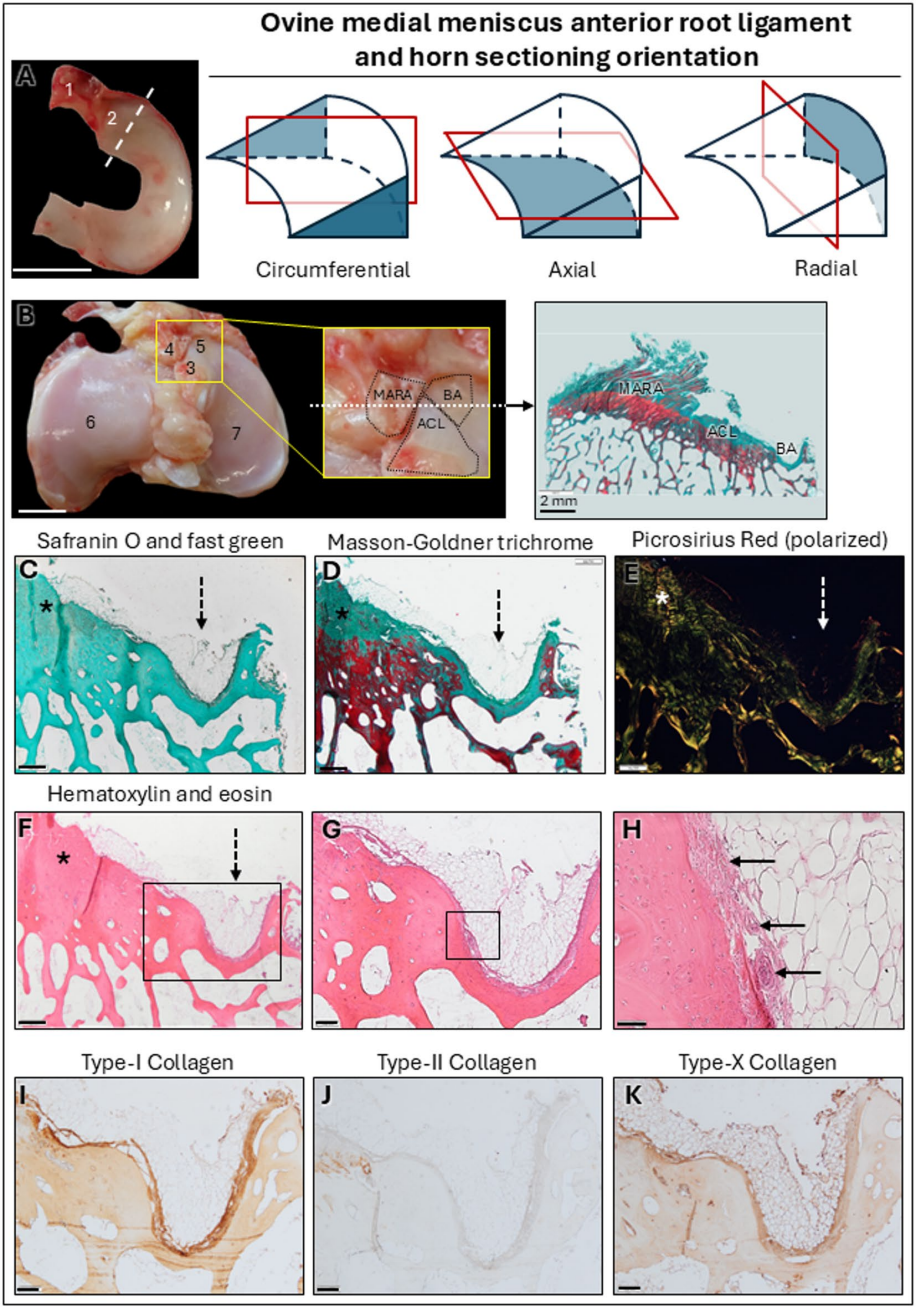
Macroscopic location and sectioning orientation of the medial meniscus anterior root (MAR) ligament, medial meniscus anterior horn (MMAH) and MAR attachment (MARA). Histological and immunohistochemical analysis of the bare area (BA) below the MAR. (**A**) Macroscopic view of the ovine medial meniscus. 1: MAR; 2: MMAH; dash-line: delimitation between meniscus horn and body. (**B**) Macroscopic view of the ovine tibial plateau; all ligaments and menisci are removed. Zoom-in: detail of structures surrounding the MARA. A white dash-line indicates the sectioning orientation of the MARA. 3: anteromedial attachment of the anterior cruciate ligament (ACL); 4: MARA; 5: BA; 6: lateral tibial plateau; 7: medial tibial plateau. (**C**–**E**) Microstructure of the bare area (dash-arrow) and the ACL (left, *). Safranin O/fast green revealed absence of proteoglycans (**C**), Masson-Goldner trichrome (**D**) showed osteoid in red, picrosirius red under polarized light (**E**) only detected birefringent fibers surrounding blood vessels, and hematoxylin eosin (**F**–**H**) facilitated the visualization of blood vessels between bone and adipose tissue (arrows). Immunostaining against type-I (**I**), type-II (**J**), and type-X (**K**) collagen showed intense immunopositivity for type-I collagen in the bone and fibers between the bone cortex and adipocytes, no immunoreactivity against type-II collagen and intense immunoreactivity against type-X collagen in the adipocyte tissue. Scale bars: **A**, **B**: 1 cm; **C**–**F**: 500 μm; **H**: 50 μm; all others: 200 μm.

Microscopically, the bare area situated between the MAR attachment (MARA), the anteromedial articular cartilage and the anteromedial bundle of the anterior cruciate ligament (ACL) (Fig. 1B) was characterized by thinner cortical bone covered by connective tissue containing mostly adipocytes and several blood vessels located between the adipocytes and the cortical bone (Fig. 1C–H). The connective tissue of the bare area exhibited strong type-I (Fig. 1I) and moderate type-X immunoreactivity (Fig. 1K). The supporting network of fibers in the connective tissue layer also displayed strong type-X collagen immunoreactivity (Table 1). Type-II collagen immunoreactivity was absent within the bare area (Fig. 1J).

**Table 1:** Semiquantitative analysis of the region-specific type-I, -II, and -X collagen immunoreactivities and vascularization, and quantitative analysis of cell density.

Region	Zone	Type-I collagen	Type-II collagen	Type-X collagen	Vascularization	Cell density
Cancellous bone		2.3 ± 0.5	0.0 ± 0.0	1.5 ± 0.5	-	854.0 ± 54.8
Enthesis	Cortical bone	2.3 ± 0.5	0.0 ± 0.0	1.5 ± 0.5	-	615.0 ± 73.9
	Calcified fibrocartilage	0.0 ± 0.0	2.3 ± 0.5	0.0 ± 0.0	0.0 ± 0.0	1,023.0 ± 10.7
	Non-calcified fibrocartilage	0.0 ± 0.0	2.8 ± 0.4	0.0 ± 0.0	0.0 ± 0.0	950.8 ± 17.3
	Adjacent ligament fibers	1.5 ± 0.5	0.0 ± 0.0	1.2 ± 0.4	1.7 ± 0.5	2,072.5 ± 54.2
Root ligament		1.7 ± 0.5	0.0 ± 0.0	1.2 ± 0.4	2.7 ± 0.4	2,090.0 ± 86.5
Medial meniscus anterior horn	Transitional line	1.5 ± 0.5	1.8 ± 0.4	1.5 ± 0.5	0.1 ± 0.5	1,618.0 ± 40.6
	Red-red zone	1.5 ± 0.5	1.5 ± 0.4	1.5 ± 0.5	1.7 ± 0.4	735.6 ± 47.5
	Red-white zone	0.5 ± 0.4	2.3 ± 0.5	1.2 ± 0.4	0.5 ± 0.5	493.3 ± 10.8
	White-white zone	0.0 ± 0.0	2.7 ± 0.5	1.2 ± 0.4	0.0 ± 0.0	608.7 ± 66.2
Bare area		2.8 ± 0.5	0.0 ± 0.0	2.8 ± 0.4	2.3 ± 0.5	491.6 ± 69.8
Connective tissue covering the root ligament		1.7 ± 0.4	0.0 ± 0.0	2.8 ± 0.5	2.8 ± 0.4	324.2 ± 53.4

Semi-quantitative differences in intensities of immunoreactivity were compared. A value of 0 reflected no reaction; 1–2, weak positivity; 2–3, moderate positivity; and 3, strong positivity. Vascularization was graded semi-quantitatively to facilitate data comprehension, where no vascularization was graded 0, low was graded 1 (1–2 blood vessels within 1 mm^2^), moderate was graded 2 (3–4 blood vessels within 1 mm^2^) and high was graded 3 (more than 5 blood vessels within 1 mm^2^). Cell density was determined as the number of cells per 1 mm^2^.

### Microstructural analysis of the MARA

**Fig. 2:**
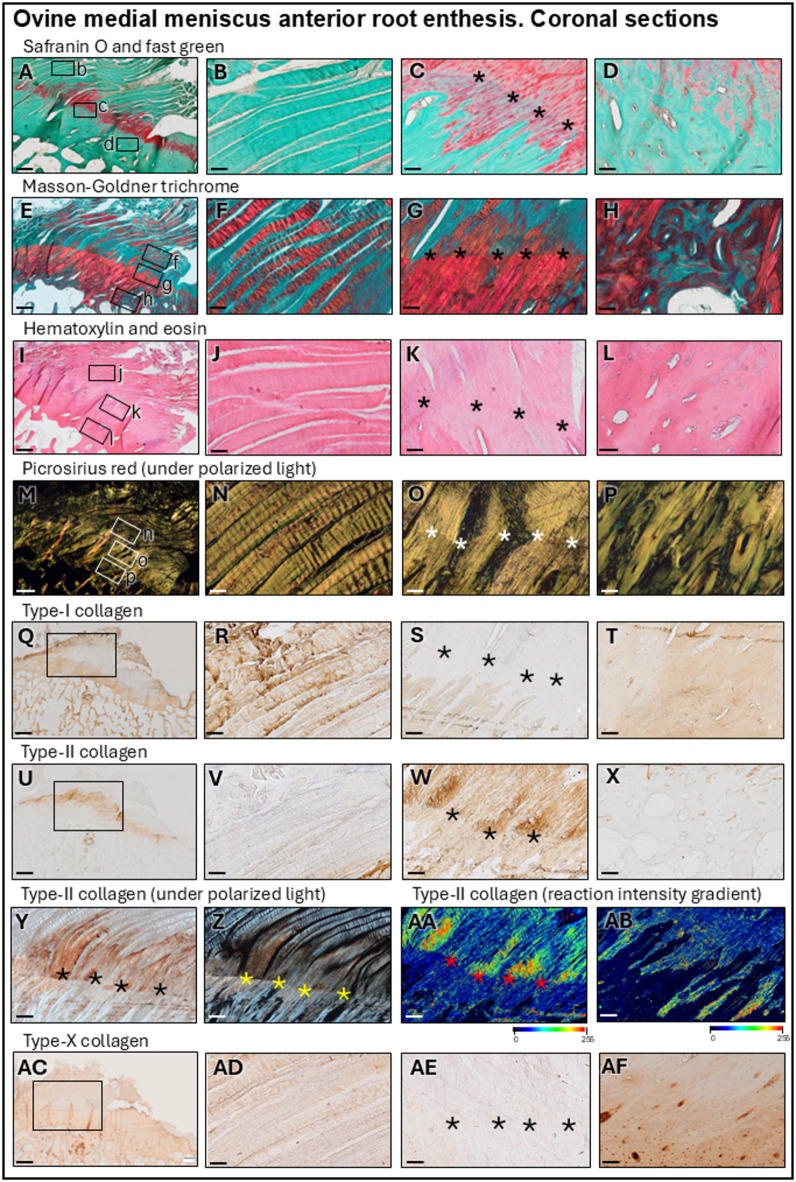
Histological and immunological staining of the ovine medial meniscus anterior root (MAR) attachment (MARA). Safranin O/fast green (**A**–**D**); fibrocartilage stained red. Masson-Goldner trichrome (**E**–**H**); fibers subjected to tension and osteoid stained red. Hematoxylin and eosin (**I**–**L**); no vascularization identified at the fibrocartilage. Picrosirius red (**M**–**P**); fiber orientation revealed by polarized light. Immunostaining against type-I (**Q**–**T**), type-II (**U**–**AB**), and type-X (**AC**–**AF**) collagen. MAR ligament fibers, proximal to the enthesis (**B**, **F**, **J**, **N**, **R**, **V**, **AD**). The fibrocartilaginous attachment of the MAR (**C**, **G**, **K**, **O**, **S**, **W**, **Y**–**AA**, **AE**) is composed of a non-mineralized and mineralized zone, separated by a tidemark (*). Bone cortex of the MAR (**D**, **H**, **L**, **P**, **T**, **X**, **AB**, **AF**). Scale bars: **A**, **E**, **I**, **M**: 500 μm; **Q**, **U**, **AC**: 2 mm; all others: 100 μm.

The matrix in the center of the enthesis stained positive for safranin O and contained mostly round fibrochondrocytes, consistent with fibrocartilage (Fig. 2A–D). In its periphery, it became negative for safranin O, thus more fibrous. The ligamentous part was negative for safranin O and did not contain fibrochondrocytes in proximity to the enthesis (Fig. 2B). Its fibrils were organized in larger weaved bundles when more distal from the enthesis (Fig. 2I,J) in a mix of tensile (Masson-Goldner: red), and compression (Masson-Goldner: green) collagen fibers (Fig. 2E-H). Their orientation was fundamentally longitudinal, as revealed by polarized light (Fig. 2M,N). A tidemark separating the calcified fibrocartilage from the non-calcified fibrocartilage was always present (Fig. 2C,G,K,O). Safranin O staining was observed in both zones (Fig. 2C). Masson-Goldner trichrome stained the non-calcified zone heterogeneously red and green, revealing a mixture of longitudinal fibers subjected to tension and compression while the calcified fibrocartilage intensely stained red, reflecting mineralization (Fig. 2G). The direction of the collagen fibers changed into a narrower angle past the tidemark (angles: 45°-90°) towards the MAR tissue (Fig. 2O). The cortical bone right below the calcified fibrocartilage was composed of osteons with different degrees of bone mineralization and without blood vessels (Fig. 2D,H,L,P).

Distribution of type-I, -II, and -X collagen immunoreactivities was location-dependent within the MARA (Table 1). Type-I collagen immunoreactivity was positive in the peripheral and distal fibers of the ligamentous part of the MARA, besides the bone (Fig. 2Q–T). The tidemark in the fibrocartilage was negative for type-I collagen (Fig. 2S). In contrast, type-II collagen immunoreactivity was exclusive and strongly positive in the fibrocartilaginous enthesis zones, both in the calcified and non-calcified fibrocartilage, localizing within the epicenter of the enthesis of the MARA (Fig. 2U–X). Polarized light revealed that the fibers with positive type-II collagen immunoreactivity in the fibrocartilage inserted into the calcified zone with a higher angle than the ones located in the non-calcified zone (Fig. 2Y,Z). Intensity gradient images for type-II collagen visualized a stronger immunoreactivity just above the tidemark (Fig. 2AA) until becoming negative in the bone just below (Fig. 2AB). Type-X collagen immunoreactivity was strongest in the thin layer of fibers that covered the ligamentous part of the MAR (Fig. 2AC–AD). The longitudinal fibrils from the ligamentous zone were slightly positive for type-X collagen (Fig. 2AD), the enthesis fibrocartilage was negative (Fig. 2AE) and the osteons were moderately positive (Fig. 2AF). The enthesis (Fig. 5A) showed type-II and absent type-I collagen immunoreactivity.

### Microstructural analysis of the root ligament

**Fig. 3:**
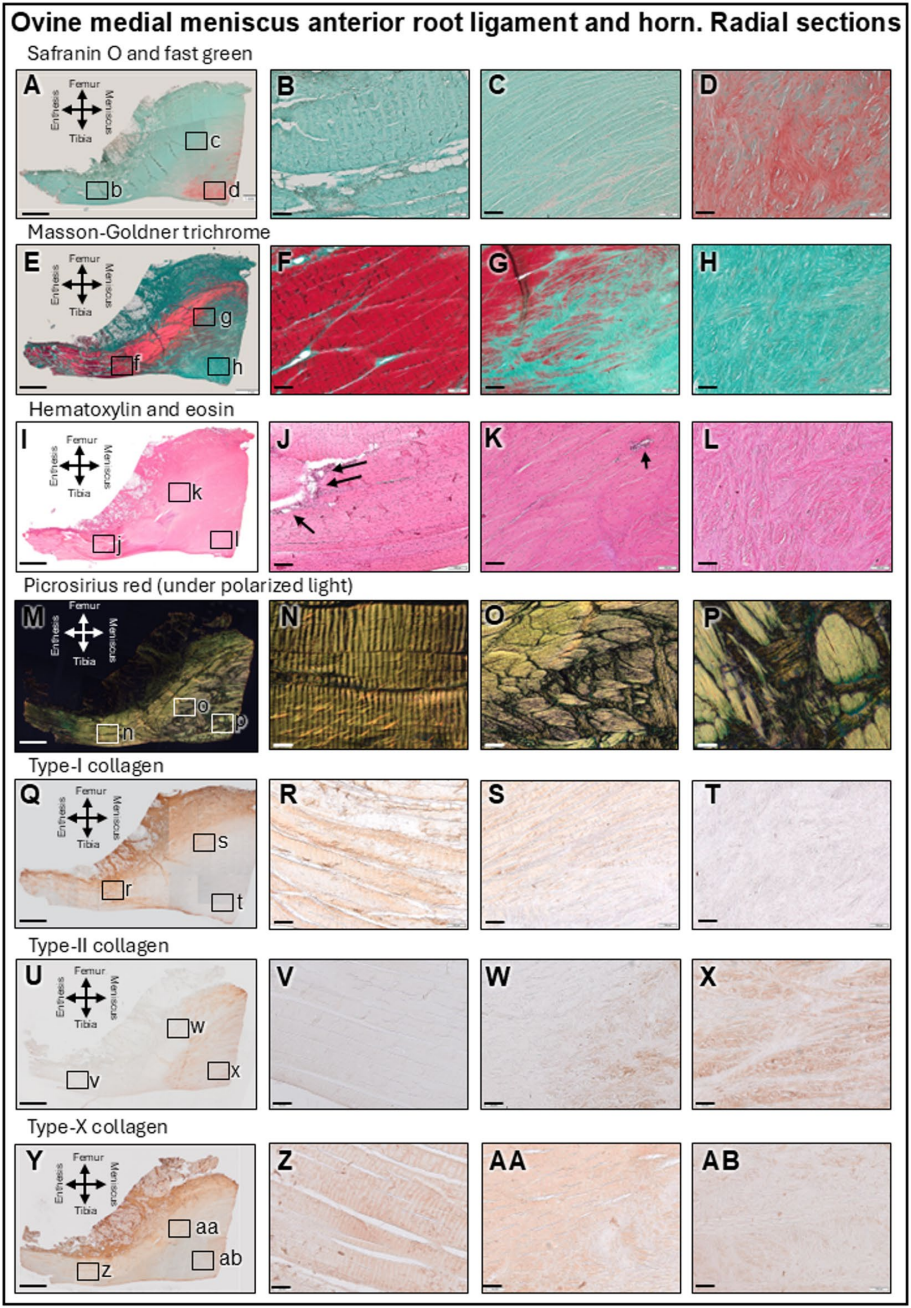
Histological and immunohistochemical analysis of radial sections of the medial meniscus anterior root (MAR) ligament and transitional line to the medial meniscus anterior horn (MMAH). Overview (**A**, **E**, **I**, **M**, **Q**, **U**, **Y**) of the root ligament (always left) and the MMAH (always right). Root ligament (**B**, **F**, **J**, **N**, **R**, **V**, **Z**) and transitional line where the fiber orientation changes (**C**, **G**, **K**, **O**, **S**, **W**, **AA**). MMAH, white-white zone (**D**, **H**, **L**, **P**, **T**, **X**, **AB**). Safranin O/fast green (**A**–**D**), white-white zone stained. Masson-Goldner trichrome (**E**–**H**), fibers subjected to tension (red) are extending into the red-red zone. Hematoxylin and eosin (**I**–**L**), blood vessels (arrows). Fiber orientation (polarized light) (**M**–**P**). Type-I collagen (**Q**–**T**), type-II collagen (**U**–**X**), and type-X collagen immunoreactivity (**Y**–**AB**). Only longitudinally sectioned fibers parallel to load forces are immunopositive. Scale bars: **A**, **E**, **I**, **M**, **Q**, **U**, **Y**: 2 mm; all others: 100 μm.

**Fig. 4:**
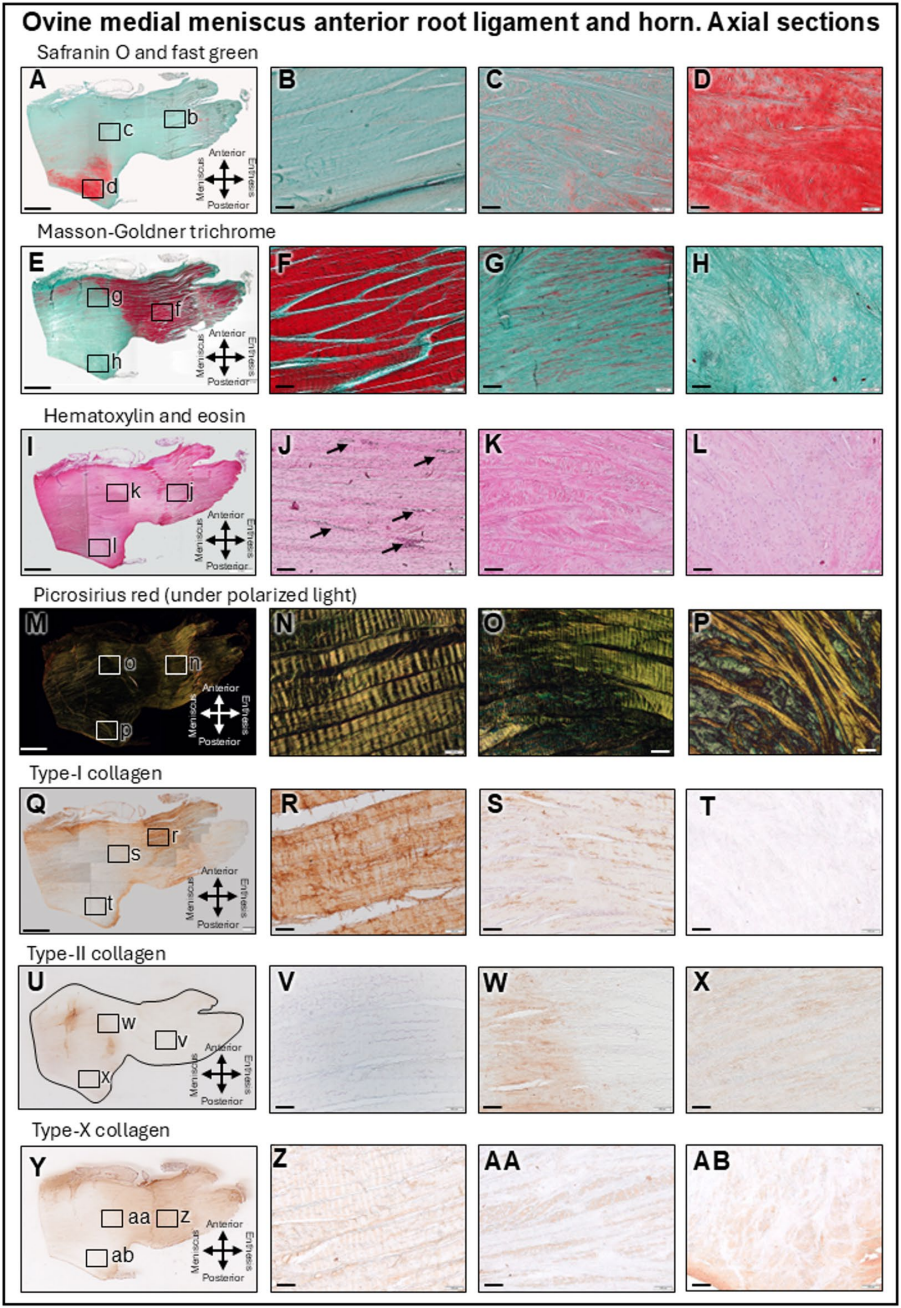
Histological and immunohistochemical analysis of circumferential sections of the medial meniscus anterior root (MAR) ligament and transitional line to the medial meniscus anterior horn (MMAH). Overview (**A**, **E**, **I**, **M**, **Q**, **U**, **Y**) of the root ligament (always right) and MMAH (always left). MAR (**B**, **F**, **J**, **N**, **R**, **V**, **Z**). Transitional line (**C**, **G**, **K**, **O**, **S**, **W**, **AA**). MMAH, white-white zone (**D**, **H**, **L**, **P**, **T**, **X**, **AB**). Safranin O/fast green (**A**–**D**) (white-white zone stained). Masson-Goldner trichrome (**E**–**H**), fibers subjected to tension (red) strikingly delimit the MAR from the horn. Hematoxylin and eosin (**I**–**L**), blood vessels (arrows). Fiber orientation (polarized light) (**M**–**P**). Type-I collagen (**Q**–**T**), type-II collagen (**U**–**X**), and type-X collagen immunoreactivity (**Y**–**AB**). Only transversally sectioned fibers are positive. Scale bars: **A**, **E**, **I**, **M**, **Q**, **U**, **Y**: 2 mm; all others: 100 μm.

**Fig. 5:**
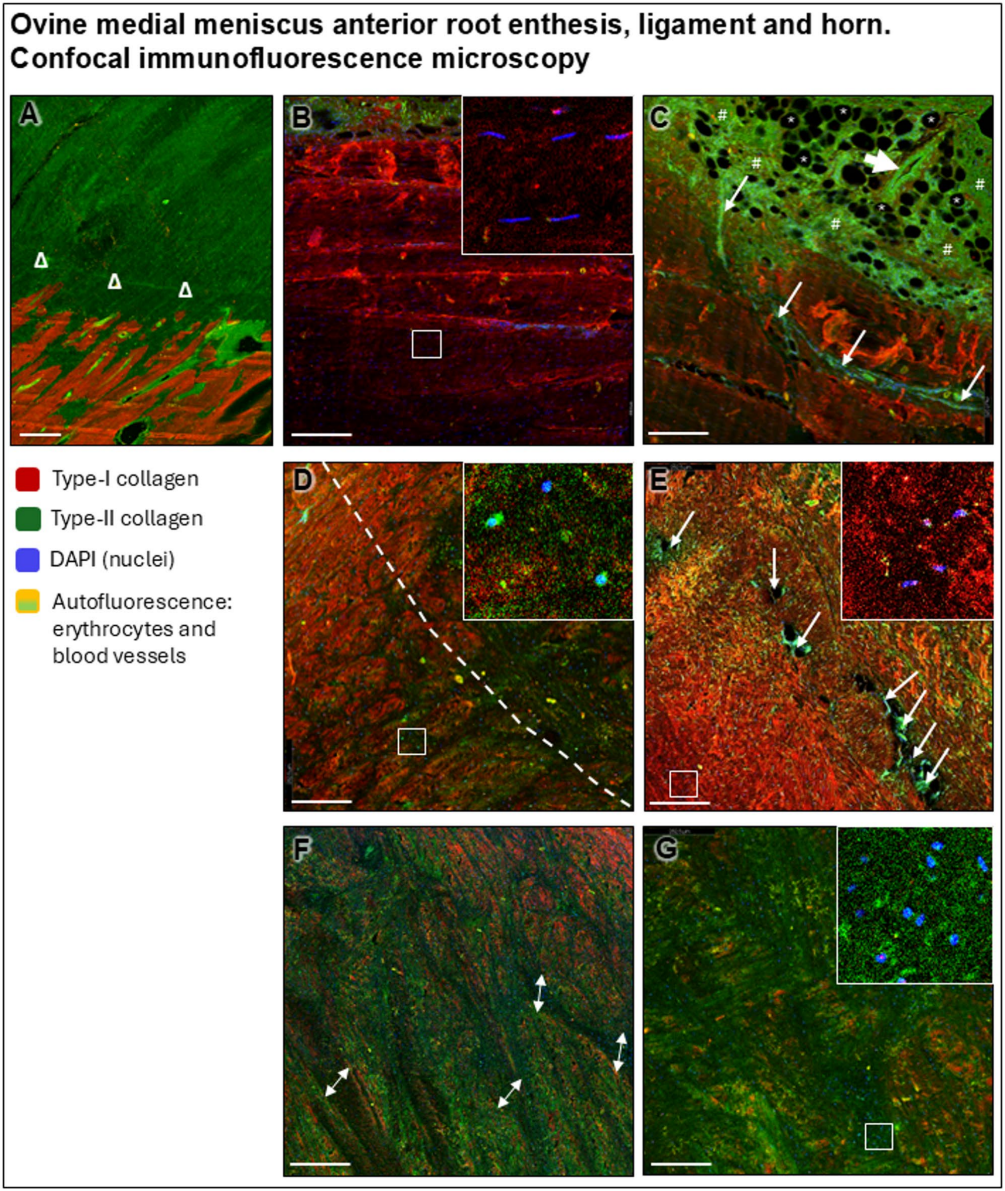
Immunofluorescence imaging with confocal microscopy of the medial meniscus anterior root (MAR) and horn (MMAH). Type-I collagen is shown in red, type-II collagen in green and nuclei in blue. (**A**) MAR attachment, where bone tissue is visibly rich in type-I collagen, and the calcified and non-calcified fibrocartilage is separated by a tidemark (Δ), both positive for type-II collagen. (**B**) MAR ligament, fascicle structure of the type-I collagen fibers are red, falciform nuclei from fibroblasts are blue, the adipose tissue appears slightly green (autofluorescence). (**C**) Detail of the vascularization of the MAR ligament. Large blood vessels (large arrow) are present in the adipose tissue. The adipose tissue is characterized by many adipocytes (droplets) that appear as empty (black) ellipsoid circles (caused by tissue processing; *) surrounded by connective tissue (green; #) and small-diameter capillaries (arrows) between the collagenous fascicles. The fibrin in the connective tissue, the blood vessels, and erythrocyte walls (yellow color when merged) all exhibit strong autofluorescence. (**D**) Transition between MAR ligament and the MMAH red-red zone (dashed line). No blood vessels from the MMAH enter the MAR ligament. Areas of type-II collagen immunoreactivity appear around fascicles of type-I collagen that changed into a radial orientation. (**E**) Red-red zone of the MMAH, exhibiting high vascularization (arrows). Higher magnification shows elliptic nuclei surrounded by type-I collagen positivity. (**F**) Red-white zone with heterogeneous immunoreactivities for type-I and -II collagen. Areas with no immunoreactivity are indicated with double-sided arrows. (**G**) White-white zone of the MMAH with predominant type-II collagen immunoreactivity. Scale bars: 200 μm.

The MAR ligament and medial meniscus anterior root horn (MMAH) were studied in the three different planes: circumferential, axial and radial (Fig. 1A). The root ligament consisted of large fascicles of longitudinal fibrils covered by connective tissue containing adipocytes and elastic fibers on its anterior and femoral sides (Figs. 3 and 4A–D). Its main ligament bundles were arranged completely longitudinally and did not intertwine with each other (Figs. 3 and 4B). Close to the MMAH they unweaved into smaller bundles that changed their orientation within the horn, where the safranin O intensity increased (Figs. 3 and 4C,D). The fibrils stained intensely red in Masson-Goldner trichrome, reflecting collagen fibers subjected to tensile forces (Figs. 3 and 4E,F). Fibroblasts were present, fibrochondrocytes absent. The MAR ligament contained several blood vessels running between the fascicles, surrounded by support fibers (Figs. 3 and 4I–K). A prominent layer of adipose tissue contained a similar amount of blood vessels. Polarized light illuminated the root ligament fascicles (stained with picrosirius red) in bright yellow until they reached the transitional line, where they unweaved and changed their direction, refracting the light as green (Figs. 3 and 4M–P). Immunoreactivity to type-I collagen was positive in the longitudinal fibril bundles and intensified in those that weaved into a network, serving to support the adipocytes and blood vessels in the connective tissue covering the ligament on its antero-femoral side (Figs. 3 and 4Q,R). The MAR ligament itself was negative for type-II collagen (Figs. 3 and 4U,V) and showed similar immunoreactivity for type-I and -X collagen (Figs. 3 and 4Y,Z) (Table 1). Immunofluorescent radial imaging of the MAR (Fig. 5B,C) revealed type-I collagen fibrils organized in fascicles. The cells in the root ligament were spindle-shaped with falciform nuclei, consistent with fibroblasts (Fig. 5B). The adipose tissue on the MAR femoral side was rich in blood vessels that divided into smaller capillaries when entering the ligament (Fig. 5C).

### Microstructural analysis of the transitional line between the root ligament and the MMAH

**Fig. 6:**
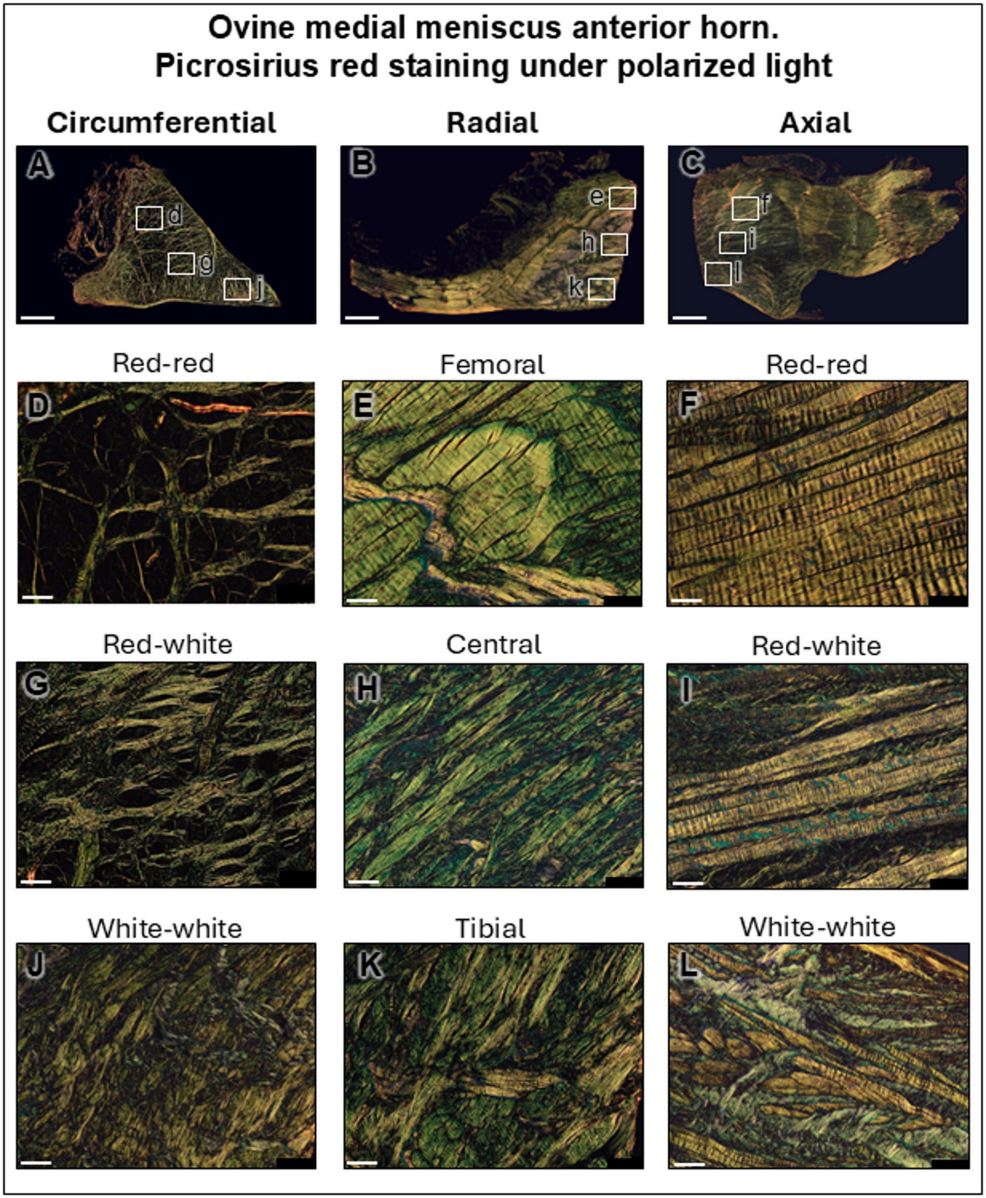
Medial meniscus anterior horn (MMAH) fiber orientation under polarized light. Under polarized light, the collagen fibers perpendicular to the light are yellow, the oblique fibers green, and the parallel ones dark. On circumferential sections (**A**, **D**, **G**, **J**), circumferential fibers cannot be detected and appear as dark spaces, where axial fibers appear yellow. The opposite pattern occurred in the axial sections (**C**, **F**, **I**, **L**). On radial sections, the radial fibers appear yellow, all others green (**B**, **E**, **H**, **K**). Scale bars: overview: 2 mm; all others: 100 μm.

In proximity to the MMAH, the fiber bundles unpacked and entwined into a fibrous network of higher complexity (Figs. 3 and 4O). The collagen fiber bundle thickness from the MAR ligament decreased by 3-fold after transitioning into the MMAH (155.8 ± 63.65 μm and 57.77 ± 29.54 μm, respectively. *P* < 0.0001). This pattern was absent in the ligamentous parts adjacent to the MAR enthesis. These fibers transitioned from their tensile-subjected characteristic into a more compression-sustained structure, although the circumferential fibrils still stained red (Masson-Goldner) while the axial ones did not (Figs. 3 and 4G). Interestingly, fibrils subjected to tensile forces further extended into the antero-femoral aspects of the MMAH. The tibial and posterior aspects of the MMAH stained positive with safranin O and contained many fibrochondrocytes (Figs. 3 and 4C,D). Here, vascularization was reduced compared with the MAR ligament (Figs. 3 and 4J,K), existing predominantly in the peripheral connective tissue. Although some vessels were identified in the outer red-red zone, none were present in the inner cartilaginous (white-white) zone (Figs. 3 and 4D,H and L). The ligament bundles were highly organized longitudinally (Figs. 3 and 4N), unweaved in the transitional line into the MMAH where they changed direction (Figs. 3 and 4O) to compound the complex collagen network in the MMAH itself (Figs. 3 and 4P). Type-I collagen immunoreactivity in the transitional line that appeared in the incoming fibers from the MAR ligament became absent after they unweaved and became part of the MMAH (Figs. 3 and 4S,T). The radial and axial fibers closer to the MMAH were strongly positive for type-II collagen (Figs. 3 and 4W,X). In the white-white zone, only radial fibers showed positive type-X collagen immunoreactivity (Fig. 3AA and AB), intersecting transversally in the circumferential plane (Fig. 4AA and AB). Thus, only these vertically-oriented fibers in the direction of the load forces of the knee exhibited positive type-X collagen immunoreactivity (Table 1). Immunofluorescence within the transitional border between the MAR and the MMAH (Fig. 5D–G) was heterogeneous. The fibers immunopositive for type-I collagen from the MAR ligament unweaved and changed direction passing the transitional border between the root ligament and MMAH (Fig. 5D), continued into the peripheral circumferential fibers of the red-red zone of the MMAH (Fig. 5E). They were absent in the white-white zone (Fig. 5G). Here, areas of absent immunoreactivity for either type-I or -II collagen appeared (Fig. 5F). Cell nuclei in the MMAH were slightly more elliptic in zones immunopositive for type-I collagen, and round in those immunopositive for type-II collagen (Fig. 5D–G). Cell densities were 2.8-fold higher in the root ligament compared with the red-red zone, 4-fold compared with the red-white zone, and 3.4-fold compared with the white-white zone (Table 1). Polarized light (Fig. 6A–C) showed that the collagen fibers in the periphery of the MMAH were arranged in large circumferential bundles, separated by axially orientated thinner fibrils that provided structural support (Fig. 6D–F). The dimensions of the circumferential bundles decreased in the middle region of the horn, reaching approximately the same proportion as the axial fibers (Fig. 6G–I). Thinner fibers with obliquus orientation and lesser organization characterized the white-white zone (Fig. 6J–L). Vascularization of the red-red zone was abundant, low in the red-white and transitional line, and absent in the white-white zone (Table 1).

**Fig. 7:**
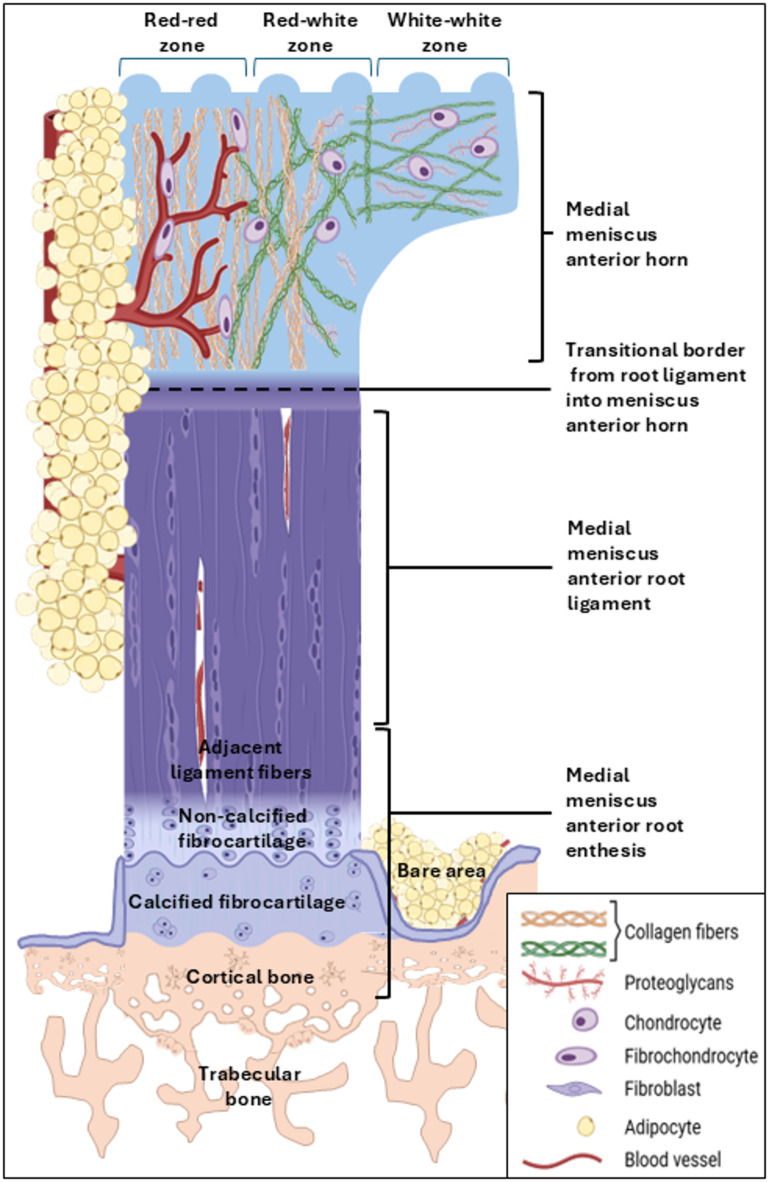
Schematic of the medial meniscus anterior root (MAR) attachment (MARA), bare area, MAR and its anterior horn (MMAH). The bare area is located between the entheses of the MAR and the articular cartilage, covered by adipocytes and blood vessels immunopositive for type-X collagen. The enthesis is composed of cortical bone, non-calcified fibrocartilage, calcified fibrocartilage, and adjacent root ligament fibers. The type-I collagen immunopositive cortical bone of the MARA is thicker than the adjacent bone. The fibrocartilage lacks vascularization and is rich in fibrochondrocytes, proteoglycans and type-II collagen. The MAR is formed of large fascicle fibers and many fibroblast-like cells, has abundant vascularization and type-I collagen fibers. The transitional line from MAR ligament into the MMAH lacks any vascularization. Here, the fascicle fibers unweave and change orientation. The MMAH red-red zone keeps the fascicle organization with circular fibers on its femoral-peripheral zone, is vascularized and contains fibrochondrocytes and type-I, -II, and -X collagen fibers. The red-white zone has a lower vascularization and contains fibrochondrocytes, with an increase of proteoglycans. The white-white zone shows a complex collagen network, lacks vascularization and is rich in type-II collagen, proteoglycans and fibrochondrocytes. Its radial fibers are immunopositive for type-X collagen. The adipose tissue that covers the MAR on its femoral and peripheral sides is rich in blood vessels surrounded by supporting type-X collagen fibers. For ease of understanding, the MAR ligament is designed without any significant curvature at the point where it attaches to the bone (the enthesis).

These findings characterize the entire ovine MAR as an enthesis organ, a term describing the enthesis as a complex structural combination of the bone-ligament attachment and its neighboring anatomical structures in order to dissipate stress concentration at the bony interface (Fig. 7). Together, the microanatomical structure of the ovine MAR is comparable to previously published findings of the human MAR (Table 2).

**Table 2:** Comparison of the microanatomical structural findings between the human and ovine medial meniscus anterior root.

	Human MAR			Sheep MAR	
	Anatomical findings (publish)	Techniques applied	References	Anatomical findings (present study)	Techniques applied
MAR enthesis	Presence of four zones Division into four zones with a visible tidemark between calcified and non-calcified fibrocartilage. Fibrochondrocytes identified in the fibrocartilage of the MAR. Absent vascularization.	Hematoxylin and eosin, Masson’s trichrome staining	^46,59^	Presence of four zones Division into four zones with a visible tidemark between calcified and non-calcified fibrocartilage. Fibrochondrocytes identified in the fibrocartilage of the MAR. They are surrounded by proteoglycans, co-localized with type-II collagen. Absent vascularization. Adjacent bare area filled with adipose tissue and blood vessels.	Hematoxylin and eosin, safranin O and fast green, immunohistoche-mistry type-II collagen
	Fiber bundle orientation Fiber bundles parallel to each other and parallel to the longitudinal axis of the ligament. Fibers in the non-calcified zone are oblique to each other. Bundle organization disappears in the calcified zone.	Scanning electron microscopy	^1^	Fiber bundle orientation Fiber bundles parallel to each other and parallel to the longitudinal axis of the ligament. Bundle organization disappears in the calcified zone of enthesis. Higher obliquity of fibers close to the enthesis. Collagen fibers oriented in different angles between calcified and non-calcified zones.	Picrosirius red visualized under polarized light, type-II collagen immunostaining visualized under polarized light and intensity gradient
	Secondary interwoven fibers Secondary interwoven fibers are present in the periphery of the MAR enthesis. They are termed “shiny white fibers” in MMPR.	Picrosirius red, toluidine blue combined with von Kossa	^28^	Secondary interwoven fibers Secondary interwoven fibers are absent in the periphery of the MAR enthesis.	Hematoxylin and eosin, picrosirius red visualized under polarized light
Root ligament	Fascicle bundle organization The fascicles are embraced by a sheath of connective tissue. Fascicles are tightly packed and oriented in parallel to each other. The membrane septae around the fascicles are composed of collagen, serving as a support structure for the fascicles. Lateral and medial posterior roots exhibit a more disorganized collagen under strain when compared to anterior roots.	Scanning electron microscopy, polarized light quantification before and after load strain	^1,45^	Fascicle bundle organization The fascicles composed of type-I collagen and fibroblasts are embraced by a sheath of connective tissue composed of type-X collagen fibers. Fascicles are tightly packed and oriented in parallel to each other. The membrane septae around the fascicles are composed of collagen, serving as a support structure for the fascicles. Fascicles mainly stain red in Masson-Goldner trichrome staining, as they are subjected to tensile forces. This pattern remarkably changes when reaching the anterior horn where the fibers stain as those subjected to compression.	Immunohistoche-mistry type-I and -X collagen, picrosirius red visualized under polarized light, Masson-Goldner trichrome staining
	Vascularization High degree of vascularization, vessels originated in the genicular artery enter from the connective tissue covering the root ligament.	India ink injected into the popliteal artery	^60^	Vascularization High degree of vascularization, vessels originated in the genicular artery from the connective tissue covering the root ligament.	Hematoxylin and eosin, confocal microscopy
Medial meniscus anterior horn	Fiber orientation The circumferential fascicles in the meniscus red-red zone are surrounded by a membrane. The fibers in the white-white zone are compact. The surface of the MMAH is composed of a collagen fiber network with a random orientation and few fibers extending in the radial direction.	Scanning electron microscopy	^1^	Fiber orientation The circumferential fascicles in the meniscus red-red zone are surrounded by a membrane and are a continuation of the fascicles in the MAR ligament. The fibrochondrocytes in the meniscus red-red zone have elliptical nuclei. The white-white zone fibers are compact and random oriented. It contains type-II collagen, fibrochondrocytes, and proteoglycan.	Safranin O and fast green, picrosirius red visualized under polarized light, immunohistoche-mistry type-II collagen
	Vascularization The red-red zone is vascularized. It receives vessels from its peripheral connective tissue that originates in the genicular artery. Vascularization in the white-white zone is absent.	India ink injected into the popliteal artery	60	Vascularization The red-red zone is vascularized. It receives vessels from its peripheral connective tissue that originates in the genicular artery. Vascularization in the white-white zone is absent.	Hematoxylin and eosin, confocal microscopy

MAR, meniscus anterior root; MMAH, medial meniscus anterior horn; MMPR, medial meniscus posterior root.

## Discussion

The most important finding of this investigation is that the region-specific microstructural complexity of the ovine MAR is largely similar to the human MAR: (1) the ovine MARA constitutes an enthesis organ together with the bare area below the root ligament that is covered by adipose tissue. (2) The ligamentous fibers of the root ligament are arranged longitudinally, in a complex network when entering the MMAH where they change their orientation the more they extend towards the white-white zone. As in humans, the MAR ligament is vascularized by blood vessels originating from the medial inferior geniculate artery, entering the ligament at its peripheral-femoral side. (3) The MMAH is of a ligamentous nature on its peripheral femoral side where collagen fascicles subjected to tensile forces extending from the root enter into it as circumferential fibers, as in humans. Only axial MMAH fibers are immunopositive for type-X collagen, highlighting its important role for load bearing. Within the three zones of the MMAH, the vascularization and shape of cells and their nuclei are consistent with the published situation in humans.

This study characterizes the microanatomy of the MAR, a key anchoring structure of the ovine medial meniscus and important musculoskeletal interface. The bare area localized in the tibia plateau and below by the MAR ligament constitutes a shallow depression of the cortical bone of the tibial plateau filled with a fat pad composed of a thin layer of adipose tissue with blood vessels below, as previously described^22^. This fat pad possibly serves as a gliding structure for the MAR ligament to avoid damage with the antero-medial insertion of the ACL and nearby cortical bone. Reflecting the theory of Benjamin and McGonagle that an enthesis with adjacent adipose tissue constitutes an “enthesis organ"^25^, postulated to disperse stress concentrations at bony interfaces^26,27^, it is likely that the adipose tissue of this bare area relates in a similar fashion to the ovine MAR. In humans, the “enthesis organ” concept has been proposed for several entheses, among which the ACL and posterior cruciate ligament, patellar and popliteal tendons^25^. All enthesis organs within a human synovial joint contain fibrocartilage, as shown also here for the ovine MARA. A similar functional adipose tissue exists posterior to the human ACL, where a fat pad also facilitates load mitigation^29^. The structural characteristics of the here described bare area filled with a fat pad would similarly aid to dissipate the stress on the MAR and enthesis itself, thus modulating the function of the meniscus root. The fat pad in the bare area potentially cushions the fibers of the MAR ligament to avoid structural damage when they exit from the bony enthesis. Here, they bend approximately 90° before further extending into the MAR ligament. Interestingly, the acute angle of the fibers in the MARA is shallower than those in the lateral meniscus anterior root attachment^28^. The direction of the collagen fibers also changes between calcified fibrocartilage, non-calcified fibrocartilage, and MAR ligament fibers. Thus, the MARA enthesis is capable of structurally adapting to the tensile forces the medial meniscus is subjected to during the motion of the knee^30^.

This work identified the complex composition of the four different enthesis zones (cortical bone, calcified fibrocartilage, non-calcified fibrocartilage, adjacent ligament fibers) in the ovine MAR to provide mobility within the tissue without localizing strain in the fibrocartilaginous interface^31^. The center of the enthesis consisted of avascular fibrocartilage that connected the root ligament to the bone, creating a barrier for both blood supply and cell-cell communication. This fibrocartilage is of paramount importance, as it reduces stress concentration and ensures that the bending of the root ligament fibers is not focused at the bone interface^26,32^, a specific characteristic thought to prevent ossification of the ligament in degenerative states^33^, highlighting its fundamental role. The collagen fibers in the fibrocartilaginous part mainly resist compressive load^34,35^ while in the root ligament they chiefly resist tension. The Masson-Goldner trichrome staining is capable of differentiating collagen fibers subjected to tension from other collagen fibers due to the differential staining with light green, which is slower to displace acid fuchsin in tensioned fibers. The different staining intensity allows to identify tensioned collagen fibers, which appear in a different shade of green or red, depending on the specific staining procedure and degree of tension^34^. The predominance of fibers stained green with Masson-Goldner trichrome in the MARA further supports its similarity to a human ligament enthesis. Its fibrocartilage was rich in type-II collagen, while type-I collagen was absent, creating a “gap phenomenon”, like in other entheses^36,37^. The fibrocartilage was positive for type-II collagen in both the calcified and non-calcified zones that were separated by a tidemark, co-localized with safranin O staining. The stronger presence of type-X collagen in fibers that cover the ligamentous zone and surrounding connective tissue in the MARA indicates the important supportive role of type-X collagen in networks of fibrils^38^. To the best of our knowledge, combined type-I, -II, and -X collagen immunoreactivities have not yet been investigated in human or sheep meniscus roots. Similar type-II collagen depositions have been described in the human medial meniscus posterior root (MMPR) enthesis^39^ and posterior horn but not in the human meniscus posterior root ligaments^40,41^. Previous studies investigated the lapine^42^ and bovine^43,44^ medial anterior and posterior root attachments. When compared with published descriptions of human meniscus roots, morphology and microstructure of both human anterior root attachments^1^, collagen fiber orientation^28,45^, and bone thickness of the MAR^46^ are all similar to the present findings in sheep (Table 2). Interestingly, human MAR fibers are more organized than MMPR fibers, a finding that may play a role in the relative paucity of human MAR tears^45^.

The here identified region-specific microstructural complexity also provides a structural basis for the mechanisms of human root injury as shown in the high prevalence of tears related to the enthesis itself or in the root ligament proximal to the enthesis, corresponding to types 2 A, 4, and 5 of the LaPrade classification^3^ (Table 3). The susceptibility of the root enthesis to tears may be explained by the striking lack of vascularization that obstructs an adequate healing response to (repetitive) microdamage, making it vulnerable to degenerative changes.

**Table 3:** Overview summary of the LaPrade classification ^3^ for human root tears and their frequency.

Classification	Definition	Frequency (from total root tears)
Type 1	Partial root tear	7%
Type 2	Complete radial root tear	67.6%
Type 2A	Tear 0–3 mm from enthesis	38%
Type 2B	Tear 3–6 mm from enthesis	16.9%
Type 2C	Tear 6–9 mm from enthesis	12.7%
Type 3	Bucket handle with complete root tear	5.6%
Type 4	Oblique tear that goes into the enthesis	9.9%
Type 5	Tear at the bone level with fracture	9.9%

The MAR ligament comprises large fascicles composed of fibers immunopositive for type-I and -X collagen. Type-I collagen is predominant in ligaments^47^, forming a quaternary structure of fibrils that specialize in mechanical properties^38^ of fundamental importance for meniscus functionality. The tensile fibers are predominantly type-I collagen immunopositive while the fibers that surrounded and packed the fascicles are predominantly type-X collagen immunopositive. The root ligament is highly vascularized, albeit lacking the shiny white fibers of both human posterior roots or the analogous supplementary fibers as described in the human MAR^48–50^. The blood vessels penetrate the root ligament from the adipose tissue that covers the enthesis on its femoral side and divide into smaller capillaries longitudinal to the MAR. The vascularization within the transition line from ligament into the MMAH was lower than the root ligament itself, suggesting that the blood vessels that supply the ligament originate directly from the perimeniscal capillary ring and not from the radial arteries within the meniscus red-red zone^51^.

Remarkably, human root tears occur less frequently within the root ligament (LaPrade types 2B and C; prevalence: ~17% and ~ 13%, resp.). Thus, it is very likely that its well-vascularized and relatively cell-rich fibers are strong and yet flexible enough to provide relative resistance to damage from acute pulling or compressing.

The transitional line between the root ligament and the fibrocartilaginous MMAH reaches a point where the large fascicles of fibers unweave and continue into the meniscal body. Especially in the peripheral femoral side, the fibers keep their longitudinal orientation. This continuity from the root to the outer or peripheral portion of the meniscus body (red-red zone), finally ending in the posterior root supports the concept of the meniscus being an extraordinary tibio-tibial ligament^43,46^. Although picrosirius red has been postulated as specific for type-I and -III collagen when exposed to polarized light^52^, our findings relate to those that establish that the color of collagen fibers stained with it under polarized light depends on collagen fiber organization, size and orientation, where large bundles of fibers perpendicular to the light appear yellow, lesser fibers with obliquus orientation are green and parallel fibers do not show birefringence and cannot be seen, independent of the collagen type^53,54^. Type-X collagen was predominantly present in fibers serving as structural support in the ligamentous part of the ovine root and in the surrounding connective tissue of the MARA, together with vertically oriented tie fibers that are parallel to the load forces in the MMAH. Their organization is comparable to (bovine) tie-fibers^43^. Cell density and vascularization in the red-red zone were high, as noted in the human meniscus^55^.

From a clinical standpoint, root tears occur rarely in the meniscus horn (LaPrade type 3; prevalence: ~6%), most likely because of the seamless transition of the strong collagen fibers of the root ligament into the horn. Of note, translational studies on the spontaneous healing capacity or surgical repair of the lapine anterior or goat posterior medial root after radial transection suggest that the structural and functional support functions may be compromised even after repair, perhaps because the attachment might heal in an elongated fashion^14,17^. During spontaneous healing, the newly formed tissue at the site of transection is characterized by scar-like loose connective tissue, glycosaminoglycans, non-bundled fibers coiled and arranged in clusters, without regular insertion of the root fibers into the bone^17^. A classical study on the surgical repair of the lapine MAR revealed that a refixated attachment matured over time from an initially highly cellular, nonspecific granulation tissue to a repair tissue that did not establish its normal insertion architecture, including the lack of a physiological distribution of types-I and -II collagen^15^. In a goat model of MMPR repair, the enthesis healed incompletely, with discontinuous fibers and a scar-like connection with partial glycosaminoglycan deposition. Similarly, the reestablishment of the connection between the root ligament and the meniscus horn remained problematic, as the collagen fiber bundles did not connect to each other, while a repair tissue surrounded the sutures^17^. Whether these and our structural findings are applicable to patients suffering from repair failure remains to be investigated^12,13^.

Limitations of this study are the lack of electron microscopy or biomechanical testing and its focus on the MAR, owing to the translational repair model^18^, although tears of the MMPR are more common. Since the same individual meniscus root cannot be histologically processed to be visualized from different points of view, comparisons between the circumferential, radial and axial planes are limited. Strengths are the accurate information on the regional distribution, providing detailed knowledge about the microstructure of the MAR enthesis, its ligamentous part and MMAH insertion. The comprehensive data of this study provides an important step towards a more detailed understanding of the regional distribution of types-I, -II, and -X collagen, proteoglycans, networks of tensile and elastic fibers, vascularization, densities and shapes of cells that is relevant when designing ovine models of root repair or replacement as it may contribute to improved repair techniques or novel tissue engineering treatments to achieve a complete healing^17,56^.

In summary, the data of the present study enhance the understanding of the ovine MARA as an “enthesis organ”. They also highlight the continuation of the MAR ligament into the MMAH where its fibers unweave, generating a complex network in the meniscus with axial support fibers that are immunopositive for type-X collagen. Together, the microanatomical structure of the ovine MAR is well comparable to that of humans, establishing it as a potential model for preclinical studies reflecting human root injury and repair.

## Conclusion

The region-specific microstructural complexity of the ovine MAR provides a structural basis to better understand its susceptibility to different root injury types and suggests that it may serve as relevant model to study the physiopathology of and therapeutic approaches to the human medial meniscus anterior root.

## Materials and methods

### Animals

24 left and right tibial plateaus from 12 skeletally mature, female Merino ewes (provided by Christian Schleich, Brunnthal, Germany) collected as part of other published^18,22^ and unpublished studies were included. Euthanasia consisted in an overdose of T-61 (6 ml / 50 kg, i.v., Intervet Deutschland, Unterschleißheim, Germany) previous sedation with xylazine (0.2 mg/kg, i.v., Elanco Tiergesundheit, Basel, Switzerland) and ketamine (20 mg/kg, i.v., Serumwerk Bernburg, Bernburg, Germany). All protocols and procedures were approved by the local governmental Animal Care Committee from Saarland University (registration number 2.4.2.2–22-2023, Saarland, Germany). All methods were conducted in accordance with the local and national legislation on the protection of animals and the NIH Guidelines for the Care and Use of Laboratory Animals. All the methodology is reported in accordance with ARRIVE guidelines.

### Histological and immunohistochemical analyses

After fixation, the 24 dissected medial meniscus root attachments were decalcified, embedded in paraffin ParaPlast X-tra (Leica Biosystems, Richmond, IL, USA), and sliced with a microtome into 3 μm coronal sections. Separately, 18 MMAHs and MARs were embedded following the same embedding process and thickness: 6 were sectioned following the circumferential plane, 6 on the radial plane, and another 6 on the axial plane. The following histological staining methods were applied: Safranin O^57^ (Carl Roth, Karlsruhe, Germany) to detect proteoglycans; HE (Carl Roth) to identify blood vessels and to determine cell density; Masson-Goldner trichrome (Morphisto, Offenbach, Germany) to differentiate between high and low mineralization in bone and between fibers subjected to tensile or compressive forces in ligaments and fibrocartilage; picrosirius red (Morphisto) under polarized light to determine fiber orientation and fiber thickness. Two additional slides from each area were incubated with primary antibodies against type-I (1:200, sc-59772; Santa Cruz Biotechnology, Dallas, TX, USA), type-II (1:50, sc-52658; Santa Cruz Biotechnology), and type-X collagen (1:100, C7974; Sigma-Aldrich, St. Louis, MO, USA) (overnight, 4 °C); then with a secondary antibody (1:200; BA-9200; Vector Laboratories, Newark, CA, USA) (1 h); with avidin-biotin-peroxidase reagent (Vectastain ABC kit; Vector Laboratories) (30 min); and finally stained with diaminobenzidine (DAB Substrate Kit, Vector Laboratories). Intensity images were obtained using a custom Python script [IF-Grad: Fluorescent image gradient converter (Version 0.1.2) (GitHub, 2022)] that converted original images into colored ones (colder colors: low intensities of the reaction, warm colors: higher intensities) to assess the degree of intensity and its localization. Semi-quantitative differences in intensity were compared between the cortical bone, calcified zone, non-calcified zone, ligament and transitional line, and adipose tissue; where value of 0 meant no reaction; 1, weak positivity; 2, moderate positivity; and 3, strong positivity. To facilitate comprehension of the different planes, MMAH fibers oriented from anterior to posterior are referred to as circumferential, from tibial to femoral as axial, and from the meniscus periphery to inner zones as radial^58^.

### Cell density, vascularization and fiber thickness quantification

Cell densities were determined as number of cells within 1 mm^2^. Vascularization was measured in a semi-quantitative fashion to facilitate data comprehension by avoiding values lower than 0.0001 of blood vessels within 1 mm^2^. Absent vascularization was graded 0, low was graded 1 (1–2 blood vessels), moderate was graded 2 (3–4 blood vessels) and high was graded 3 (more than 5 blood vessels) within 1 mm^2^. The collagen fiber bundle thickness was measured perpendicular to its longitudinal orientation at 50-µm intervals. Images were taken with an Olympus SC50 Camera and CellSens Software (v1.18 Standard; Olympus, Tokyo, Japan) and processed with ImageJ 1.54p (National Institutes of Health, Bethesda, MD, USA). Polarized light images were obtained with the same camera (Olympus SC50) and the BX45-PO polarizer (Olympus) in combination with a U-ANT nosepiece analyzer (Olympus), perpendicularly to the tissue sample. Objective lenses utilized were 2x for mosaic overview images and at 4x to study the bare area, all others being taken at 10x.

### Confocal microscopic immunofluorescent analysis

MAR entheses and radial-sectioned MMAHs (*n* = 3 each) were selected for immunofluorescence; incubated simultaneously overnight with primary antibodies against type-I (1/200, sc-59772; Santa-Cruz Biotechnology) and type-II collagen (1/200, n.1280; Novus Biologicals, Centennial, CO, USA) (4 °C), then simultaneously incubated with an anti-rabbit IgG conjugated with AlexaFluor488 (1/800, ab150077; Abcam, Cambridge, United Kingdom) and an anti-mouse IgG conjugated with AlexaFluor647 (1/800, ab150115; Abcam) (2 h) and with DAPI (62248; Invitrogen by Thermo Fisher Scientific, Waltham, MA, USA) (10 min). Images were taken with a white laser confocal microscope (Leica STELLARIS DMI8; Leica, Wetzlar, Germany). Excitation laser configuration was 405 nm (DAPI), 488 nm (AlexaFluor488), and 647 nm (AlexaFluor647). The emission detector was set to the following channels: 420–488 nm (DAPI), 504–574 nm (AlexaFluor488), and 649–715 nm (AlexaFluor647). All microscopic pictures taken had a Z-stack of 2.5 μm thickness and were processed using LAS X software version 4.6.1.27508 (Leica).

### Statistical analysis and additional software

Semiquantitative and quantitative data are given as mean and standard deviation. All statistical analysis were performed with Prism v.10.2.3 (GraphPad, San Diego, CA, USA). Figure 7 was created with Microsoft Office PowerPoint (Microsoft, Redmond, WA, USA) and models from Biorender.com (Carretero, M. (2025) https://BioRender.com/c4f8ceo).

## Data Availability

The data that support the findings of this study are available from the corresponding author upon reasonable request.
